# Triple-Negative Essential Thrombocythemia: Clinical-Pathological and Molecular Features. A Single-Center Cohort Study

**DOI:** 10.3389/fonc.2021.637116

**Published:** 2021-03-12

**Authors:** Daniele Cattaneo, Giorgio Alberto Croci, Cristina Bucelli, Silvia Tabano, Marta Giulia Cannone, Gabriella Gaudioso, Maria Chiara Barbanti, Kordelia Barbullushi, Paola Bianchi, Elisa Fermo, Sonia Fabris, Luca Baldini, Umberto Gianelli, Alessandra Iurlo

**Affiliations:** ^1^Hematology Division, Foundation IRCCS Ca' Granda Ospedale Maggiore Policlinico, Milan, Italy; ^2^Department of Pathophysiology and Transplantation, University of Milan, Milan, Italy; ^3^Division of Pathology, Foundation IRCCS Ca' Granda Ospedale Maggiore Policlinico, Milan, Italy; ^4^Laboratory of Medical Genetics, Foundation IRCCS Ca' Granda Ospedale Maggiore Policlinico, Milan, Italy; ^5^Dermatology Unit, Foundation IRCCS Ca' Granda Ospedale Maggiore Policlinico, Milan, Italy; ^6^Department of Oncology and Hemato-Oncology, University of Milan, Milan, Italy

**Keywords:** essential thrombocytemia, triple-negative, bone marrow morphology, next-generation sequencing, prognosis

## Abstract

Lack of demonstrable mutations affecting *JAK2, CALR*, or *MPL* driver genes within the spectrum of *BCR-ABL1*-negative myeloproliferative neoplasms (MPNs) is currently referred to as a triple-negative genotype, which is found in about 10% of patients with essential thrombocythemia (ET) and 5–10% of those with primary myelofibrosis (PMF). Very few papers are presently available on triple-negative ET, which is basically described as an indolent disease, differently from triple-negative PMF, which is an aggressive myeloid neoplasm, with a significantly higher risk of leukemic evolution. The aim of the present study was to evaluate the bone marrow morphology and the clinical-laboratory parameters of triple-negative ET patients, as well as to determine their molecular profile using next-generation sequencing (NGS) to identify any potential clonal biomarkers. We evaluated a single-center series of 40 triple-negative ET patients, diagnosed according to the 2017 WHO classification criteria and regularly followed up at the Hematology Unit of our Institution, between January 1983 and January 2019. In all patients, NGS was performed using the Illumina Ampliseq Myeloid Panel; morphological and immunohistochemical features of the bone marrow trephine biopsies were also thoroughly reviewed. Nucleotide variants were detected in 35 out of 40 patients. In detail, 29 subjects harbored one or two variants and six cases showed three or more concomitant nucleotide changes. The most frequent sequence variants involved the *TET2* gene (55.0%), followed by *KIT* (27.5%). Histologically, most of the cases displayed a classical ET morphology. Interestingly, prevalent megakaryocytes morphology was more frequently polymorphic with a mixture of giant megakaryocytes with hyperlobulated nuclei, normal and small sized maturing elements, and naked nuclei. Finally, in five cases a mild degree of reticulin fibrosis (MF-1) was evident together with an increase in the micro-vessel density. By means of NGS we were able to identify nucleotide variants in most cases, thus we suggest that a sizeable proportion of triple-negative ET patients do have a clonal disease. In analogy with driver genes-mutated MPNs, these observations may prevent issues arising concerning triple-negative ET treatment, especially when a cytoreductive therapy may be warranted.

## Introduction

Essential thrombocythemia (ET), together with polycythemia vera (PV) and primary myelofibrosis (PMF), belongs to the so-called “classic” *BCR-ABL1*-negative myeloproliferative neoplasms (MPN) category. It is mainly characterized by an increased platelet production and sustained thrombocytosis which leads to an increased risk of thrombo-hemorrhagic complications (1, 2). It is the most common *BCR-ABL1*-negative MPNs, with an annual incidence estimated at 0.38–1.7 ×10^5^ persons (3), a reported median age at presentation of 68 years and a female-to-male ratio of 2:1 (4).

Essential thrombocythemia diagnosis is currently made according to the 2017 WHO criteria and is based on a composite assessment of clinical-laboratory features, including also molecular analyses (5). Indeed, in 2005 the *JAK2*V617F mutation has been reported to play a crucial role in the pathogenesis of these disorders, being identifiable in about 50–60% of ET patients (6–9). Furthermore, this mutation seems to be associated with peculiar phenotypes in the different *BCR-ABL1*-negative MPNs: specifically, in ET its presence is consistently associated with older age at diagnosis, higher hemoglobin levels, higher white blood cells counts, and lower platelet counts; it also clusters with a lower risk of fibrotic evolution and a higher risk of thrombosis (10–14).

Subsequently, mutations in genes other than *JAK2* have been described: *MPL* mutations, with a frequency ranging from 3 to 8% (15, 16), and, more recently, somatically acquired mutations in the calreticulin (*CALR*) gene, reported in about 25–35% of all ET cases (17, 18).

The clinical course of *CALR*-mutated ET patients was reported to be better than in the presence of the *JAK2*V617F mutation, as subjects with *CALR* mutations seem to suffer from a lower risk of thrombosis when compared with both *JAK2*- and *MPL*-mutated cases, with a favorable impact on thrombosis-free survival (TFS) (19–23).

Lack of demonstrable mutations affecting the above-mentioned driver genes is currently referred to as a triple-negative genotype, which is found in about 10% of patients with ET and 5–10% of those with PMF. A few triple-negative ET cases have been shown to carry activating mutations of *MPL* gene outside exon 10, and these non-canonical mutations may be either somatically acquired or inherited (24, 25). Therefore, these subjects may have a true *BCR-ABL1*-negative MPN associated with non-canonical *MPL* mutations, or, alternatively, hereditary thrombocytosis (26).

Very few papers are presently available on triple-negative ET, which is basically described as an indolent disease with a low incidence of vascular events, differently from triple-negative PMF, which is an aggressive myeloid neoplasm, with a significantly higher risk of leukemic evolution (27, 28). More specifically, in WHO-defined ET, survival is near-normal (15-year survival of about 80%) and similar to that of the sex- and age-standardized European population (29), and the 10-year risk of MPN – blast phase (MPN-BP) or post-ET myelofibrosis (PET-MF) evolution is <1% (29–32).

Accordingly, triple-negative ET differential diagnosis is of crucial relevance, particularly vs. reactive thrombocytosis and, most notably, pre-fibrotic PMF, as the latter condition significantly impacts on patients' management due to its increased risk of fibrotic progression/leukemic evolution and reduced survival (29).

The advent of next-generation sequencing (NGS) technologies has greatly improved the understanding of MPN pathogenesis. Molecular profiling of triple-negative ET, using targeted gene panels or whole-exome sequencing, revealed the presence of so-called “non-driver” mutations. These variants may chronologically precede or follow the acquisition of driver mutations, thus conferring a selective advantage to neoplastic cells, and affect genes involved in epigenetic regulation (*TET2, DNMT3A, ASXL1, EZH2*, and *IDH1/2*), RNA splicing (*SF3B1, SRSF2*, and *U2AF1*) and regulation of cytokine signaling (*CBL*) (25, 33). Although mutations in these genes are not restricted to MPNs, their presence can be of diagnostic value in the triple-negative ET cohort, thus providing evidence of clonality (25, 33).

The aim of the present study was to evaluate the bone marrow (BM) morphology and the clinical-laboratory parameters of ET patients, defined upon strict adherence to the 2017 WHO criteria and featuring a triple-negative genotype, and to determine their molecular profile by means of a targeted NGS panel of current clinical use, in order to identify potential clonal biomarkers.

## Materials and Methods

### Patients

The present single-center cohort study involved a consecutive series of 40 patients, regularly followed up at the Hematology Unit of our Institution between January 1983 and January 2019, who received a diagnosis of triple-negative ET according to the 2017 WHO classification criteria. Inclusion criteria were the availability of demographic, clinical, hematologic, and histologic data at diagnosis, stored BM, and at least one granulocyte DNA sample (collected at the time of *JAK2* mutational screening) to perform NGS analysis. All patients lacked mutations in *JAK2, CALR*, or *MPL* genes, as routinely assessed by allele-specific PCR or Sanger sequencing and confirmed after deep targeted NGS analysis (see below). Baseline clinical characteristics and outcome measures (thrombosis, hemorrhages, PET-MF and MPN-BP, death, and overall and thrombosis-free survival) were evaluated. We considered as main cardiovascular risk factors the presence of arterial hypertension, diabetes mellitus, hyperlipidemia, and smoking attitude. Events of major thrombosis and bleeding were recorded when objectively documented and according to standard definitions as described in detail by the Cytoreductive Therapy in Polycythemia Vera Collaborative Group (34). Diagnosis of PET-MF was made in accordance with the International Working Group for Myeloproliferative Neoplasms Research and Treatment (IWG-MRT) criteria (35). Diagnosis of MPN-BP was made with a 20% BM or peripheral blood blast threshold (36). The patients were treated with antiplatelet and cytoreductive agents (hydroxycarbamide and anagrelide) according to institutional guidelines which were based on current recommendations [the Italian guidelines after 2004 (37) and the European LeukemiaNet guidelines after 2011 (38, 39)]. Thrombosis-free survival was measured from the date of diagnosis to the date of the first documented major thrombosis. Follow-up information was updated in August 2020.

### Methods

#### Molecular Analyses

The *JAK2*V617F mutation was detected by allele-specific PCR according to the protocol of Baxter et al. (7) and confirmed by direct Sanger sequencing. Quantitative analysis of the allele burden of the *JAK2*V617F mutation was performed by RQ-PCR using JAK2 MutaQuant (Ipsogen Inc, New Haven, CT, USA). The cut-off used for defining a case as negative for *JAK2*V617F mutation was 0.5%.

*MPL* mutations, in particular W515L, W515K, W515A, S505N, and G509C, were tested by direct sequencing of exon 10. Primer used were: MPL10F 5' TAGCCTGGATCTCCTTGGTG 3'; MPL10R 5' CCTGTTTACAGGCCTTCGGC 3'.

Mutations in exon 9 of the *CALR* gene were also assessed by the bidirectional sequencing approach as previously described (18). All sequencing analyses were performed on ABI PRISM 310 Genetic Analyzer (Applied Biosystems, Warrington, UK) using the Big Dye Terminator v1.1 Cycle Sequencing Kit (Applied Biosystems, Warrington, UK).

Next-generation sequencing analysis was performed using DNA extracted from peripheral blood granulocytes by the Ampliseq Myeloid Panel (Illumina), a commercially available NGS panel of current use in specialized laboratory for clinical practice, which allows sequencing of 40 genes mostly involved in myeloid malignancies (Supplementary Table 1). Genomic libraries were prepared using 20 ng of gDNA template and sequenced following Illumina's standardized protocol. The final amplicon pool of 14 samples/run was loaded at 9 pM on MiSeq System (v3 chemistry). The sequencing was performed sequentially from both ends each for 151 cycles. Obtained sequences were aligned to the reference genome (GRCh37/hg19) using MiSeq Reporter software (Illumina), which detected discrepancies among samples and reference genomes and determined their type, such as deletions, insertions, and SNPs. We considered data showing the following parameters acceptable for analyses: sequencing coverage >100X (bi-directional true paired-end sequencing), quality of 100, and a variant frequency (VAF) ≥ 3%. Concerning NGS data, a minimum quality score of Q30 (corresponding to a 1:1,000 error rate) was required for a minimum of 90% of bases sequenced and we excluded runs failing these metrics. The cluster density of each run was 600/800 Mean unique depth of coverage across the capture region of 3,000 amplicons.

To annotate sequence variants, Illumina Base Space Sequence Hub software was used. We included in our analysis: (1) low frequency single nucleotide variants, predicted as pathogenic by at least one of the following interpretation tools: FATHMM-MKL (http://fathmm.biocompute.org.uk/), SIFT (https://sift.bii.a-star.edu.sg/), DANN (https://cbcl.ics.uci.edu/public_data/DANN/), Poly-phen 2 (http://genetics.bwh.harvard.edu/pph2/); (2) single nucleotide variants with a minor allele frequency ≥ 0.01 (MAF ≥ 1%) predicted as pathogenic by at least one of the same tools and located in genes already described as associated with MPNs; (3) insertion/deletion variants, reported as pathogenic/likely pathogenic by clinical databases (Supplementary Table 2).

#### Cytogenetic Analyses

G-banding with trypsin performed on fresh BM aspirates was the standard technique for chromosome analysis with at least 20 metaphases described (40). Normal karyotype was defined as the absence of any chromosomal abnormality in a minimum of 20 metaphases examined. Chromosomal abnormalities were considered clonal if the same structural abnormality or extra chromosome appears in at least two and monosomy in at least three metaphases. A complex karyotype was defined as the presence of three or more distinct structural or numeric abnormalities. Monosomal karyotype was defined as two or more distinct autosomal monosomies or single autosomal monosomy associated with at least one structural abnormality.

#### Bone Marrow Biopsy

Histologic confirmation of the clinical diagnosis of ET as defined by the 2017 WHO classification was performed by two experienced pathologists (G.A.C and U.G.). Formalin-fixed, paraffin-embedded BM biopsy samples obtained at diagnosis were available for all patients. Sections were stained with hematoxylin-eosin, Giemsa, and Gomori's silver impregnation for evaluation of morphologic features and BM fibrosis. Immunohistochemistry for the evaluation of CD34+ precursors and micro-vessel density was performed via automated Dako OMNIS system, by means of the streptavidin-biotin-peroxidase-conjugated method.

Morphologic parameters were assessed according to the WHO classification, as follows: age-related cellularity, quantitative assessment of granulopoiesis, erythropoiesis and megakaryocytes, left-shifting of granulopoiesis and erythropoiesis (relative increase in immature to intermediate forms or proerythroblasts), presence, size (small vs. large) and density (loose vs. dense) of megakaryocyte clusters, morphologic features of megakaryocytes (hyperlobulated nuclei, cloud-like/bulbous nuclei, naked nuclei, maturation defects), grade of BM fibrosis, presence of osteosclerosis, density of vessels, presence of vascular ectasia and endoluminal hemopoiesis, presence of reactive lymphoid aggregates.

## Results

### Clinical Characterization

After NGS confirmation, 40 patients with triple-negative ET, diagnosed from January 1983 to January 2019, were included in this study. Their clinical-laboratory features at diagnosis are reported in Table 1. Median age at diagnosis was 50.2 years (range, 20–85) and 60.0% of the patients were females.

**Table 1:** Baseline clinical and laboratory features of 40 triple-negative ET patients.

	**Patients (*n* = 40)**
Male/female	16/24
Age (years), median (range)	50.2 (20–85)
Hb (g/dl), median (range)	13.8 (10.8–16.1)
Hct (%), median (range)	41.8 (33.2–49.1)
WBC count (×10^9^/L), median (range)	8.01 (5.23–12.5)
PLT count (×10^9^/L), median (range)	595 (443–2456)
LDH (IU/L), median (range)	220 (105–462)
Serum erythropoietin (mIU/mL), median (range)	7.15 (0.6–14.1)
Circulating CD34+ cells (/μl), median (range)	5 (1–9)
Cytogenetic abnormalities, *n* (%)	7 (17.5)
Palpable splenomegaly, *n* (%)	5 (12.5)
Previous thrombosis, *n* (%)	2 (5.0)
Previous hemorrhages, *n* (%)	/
**Conventional thrombotic score**, ***n*** **(%)**	
Low risk	25 (62.5)
High risk	15 (37.5)
**IPSET-Thrombosis**, ***n*** **(%)**	
Low risk	28 (70.0)
Intermediate risk	10 (25.0)
High risk	2 (5.0)
**Revised IPSET-Thrombosis**, ***n*** **(%)**	
Very Low risk	26 (65.0)
Low risk	/
Intermediate risk	12 (30.0)
High risk	2 (5.0)
Cytoreductive therapy, *n* (%)	22 (55.0)
Antiplatelet therapy, *n* (%)	37 (92.5)
Follow-up (years), median (range)	7.0 (1.2–36.8)
Deceased, *n* (%)	4 (10.0)

*ET, essential thrombocythemia; Hb, hemoglobin; Hct, hematocrit; WBC, white blood cells; PLT, platelets; LDH, lactate dehydrogenase*.

After a median duration of follow-up of 7.0 years (range, 1.2–36.8), no case of fibrotic transformation or leukemic evolution was recorded. On the contrary, myelodysplastic syndrome (MDS) was diagnosed in one (2.5%) patient after more than 32 years of follow-up.

After a median time from diagnosis of 9.9 years (range, 4.4–15.4), two (5.0%) patients experienced an arterial thrombosis (acute myocardial infarction and ischemic stroke in one case each). Instead, no case of venous thrombosis or significant hemorrhage was reported. Four (10.0%) subjects had died and only in two cases (5.0%) the cause of death was defined as ET-related.

As far as the treatment of these patients is concerned, hydroxycarbamide (20 patients, 50.0%) was the primary cytoreductive agent used, followed by anagrelide (two patients, 5.0%). Sixteen (40.0%) cases were on antiplatelet therapy alone, whereas two (5.0%) patients received no specific therapy.

### Cytogenetics

As reported in Table 1, cytogenetic data were available at diagnosis in all patients, with a normal karyotype being reported in most of the cases (33/40, 82.5%). Specifically concerning chromosomal abnormalities, they were more frequently represented by sexual chromosome abnormalities (-Y or -X, 5/7), with +8 detected in the remaining two cases. No complex or monosomal karyotype was reported. Interestingly, the single case who evolved into MDS showed a normal karyotype at diagnosis and maintained it during follow-up.

### Molecular Characterization

**Figure 1:**
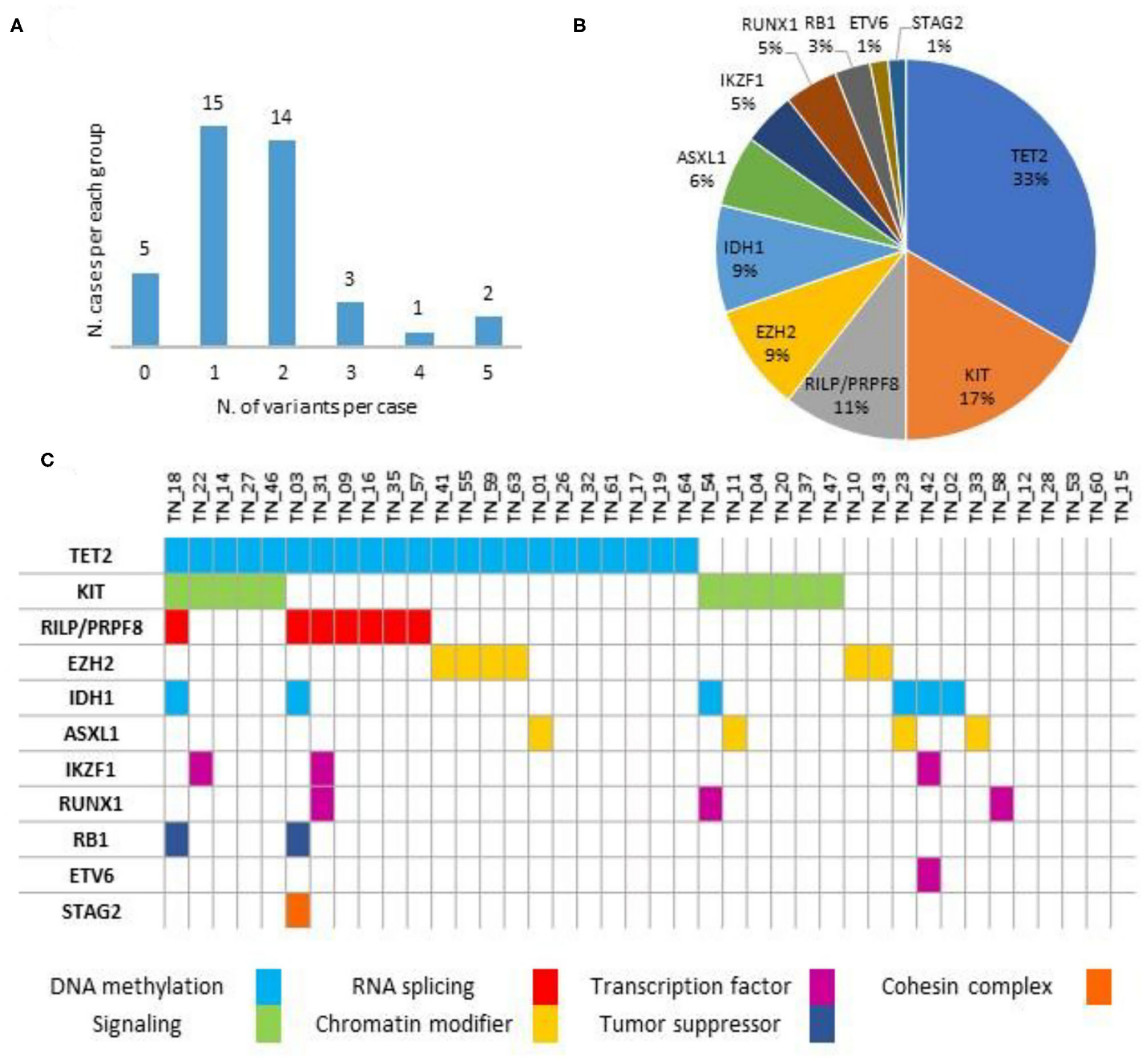
Variants distribution **(A)**, relative frequencies of pathogenic variants **(B)** and mutational spectrum **(C)** along the series (*n* = 40).

As detailed in Figure 1 and Supplementary Table 2, no variants were detected in five (12.5%) cases, with the remaining 35 (87.5%) patients harboring at least one likely damaging variant (i.e., VUS excluded). In detail, most of the subjects (29/40, 72.5%) harbored one or two mutations, whereas the remaining six (15.0%) showed three or more concomitant variants. No specific mutational pattern was identified. The most frequent sequence variants involved the *TET2* gene (22/40, 55.0%), followed by *KIT* (11/40, 27.5%). Other variants detected in at least one case also include *EZH2, RILP/PRPF8, IDH1, ASXL1, IKZF1, RUNX1, RB1, ETV6*, and *STAG2*. Notably, VAF was observed in a 0.03/0.98 range, as detailed in Supplementary Table 2.

### Histopathologic Characterization

**Figure 2:**
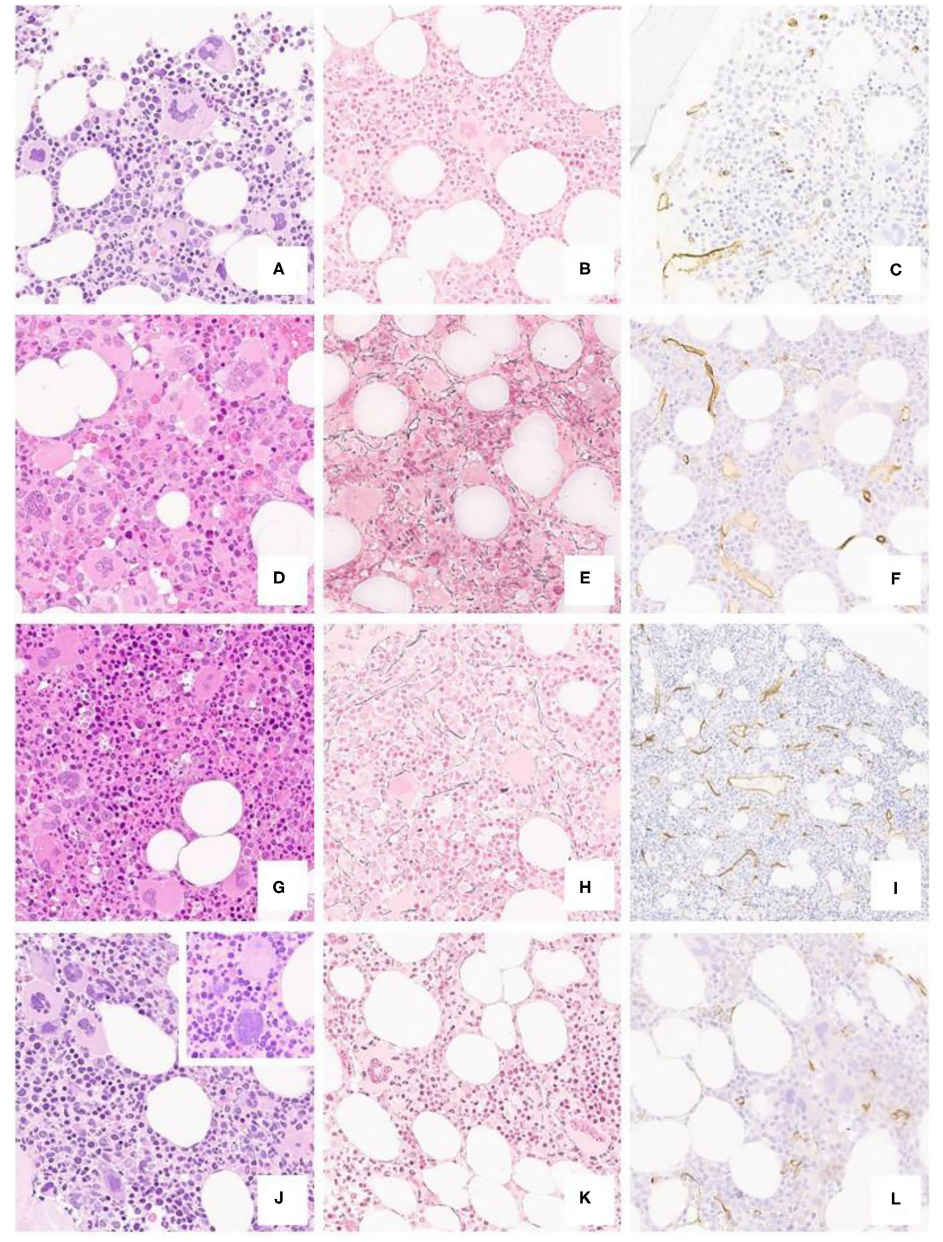
Typical ET case from a 40-year-old patient (**a** H/E, 20x), featuring a polymorphic spectrum of megakaryocytes, but with giant, hyperlobated forms and scant naked nuclei, MF-0 fibrosis (**b** Gomori, 20x) and unremarkable CD34+ blasts and vessels (**c** CD34, 20x). BM biopsy from a 35-year-old subject (**d** H/E, 20x) featuring mild hypercellularity, increased, left-shifted erythropoiesis with proerythroblasts and absolute predominance of giant, hyperlobated megakaryocytes; there is only a minor increase in reticulin fibers (**e** Gomori, 20x) and vessel density (**f** CD34, 20x). A further case from a 31-year-old patient shows mild panmyelosis (**g** H/E, 20x) with polymorphic megakaryocyte clusters, but featuring ET-like giant forms, along with dim reticulin fibers increase (**h** Gomori, 20x) and mild micro-vessel proliferation (**i** CD34, 20x). The last panel from a 52-year-old individual depicts a normocellular bone marrow (**j** H/E, 20x) with clustering of predominantly hyperlobulated megakaryocytes, with scant bulbous forms (inset) or with maturation defects, with unremarkable fibrosis and CD34+ positive cells (**k** Gomori, 20x; **l** CD34, 20x).

Morphologic features are detailed in Table 2 and Figure 2. In brief, most of the cases displayed a normocellular BM with increased number of megakaryocytes with the classical giant forms with hyperlobulated (“staghorn”) nuclei.

**Table 2:** Histologic features of 40 triple-negative ET patients.

**Histologic features**	***N*. cases (%) (*n* = 40)**	**Frequency of features in ET according to WHO 2017**
**Cellularity**		
Age related increase	5/40 (12.5%)	10–19%
**Granulopoiesis**		
Increased in quantity	3/40 (7.5%)	<10%
Left shifted	5/40 (12.5%)	<10%
**Erythropoiesis**		
Increased in quantity	10/40 (25%)	<10%
Left shifted	16/40 (40%)	<10%
**Myeloid erythroid ratio**		
<3:1	13/40 (32.5%)	NA
=3:1	26/40 (65%)	
>3:1	1/40 (2.5%)	
**Megakaryopoiesis**		
Increased in quantity	40/40 (100%)	>80%
**Clusters**		
Small cluster (>3 cells)	40/40 (100%)	10–19%
Large cluster (>7 cells)	0/40 (0%)	0%
Loose cluster	40/40 (100%)	20–49%
Dense cluster	11/40 (27.5%)	<5%
**Size of cells**		
Normal/small	37/40 (92.5%)	<5%
Giant	40/40 (100%)	20–49%
**Morphology of cells**		
Hyperlobulated nuclei	40/40 (100%)	50–80%
Cloud-like/bulbous nuclei	2/40 (5%)	<10%
Naked nuclei	17/40 (42.5%)	20–49%
Maturation defects	4/40 (10%)	0%
**Prevalent morphology**		
Giant, hyperlobulated	17/40 (42.5%)	NA
Polymorphic	23/40 (57.5%)	NA
**Fibrosis (WHO)**		
MF 0	34/40 (85%)	<5%
MF 1	5/40 (12.5%)	0%
MF 2-3	0/40 (0%)	0%
Osteosclerosis	0/40 (0%)	
**CD34+** **vessels**		
Increased density	5/40 (12.5%)	NA
Ectasic vessels	16/40 (40%)	NA
Endoluminal hemopoiesis	1/40 (2.5%)	NA
**Lymphoid aggregates**		
Present	8/40 (20%)	<5%

*ET, essential thrombocythemia; NA, not assessed*.

In 10 (25%) out of 40 patients the erythropoietic series resulted variably increased with some degree of left-shifting (16/40, 40%) and a reduction of the myeloid/erythroid ratio (13/40, 32.5%). On the contrary, a slight increase of the granulopoietic series was found in three cases (3/40, 7.5%) with left-shifting (5/40, 12.5%).

Megakaryocytes prevalent morphology was polymorphic in more than half of the subjects (23/40, 57.5%) with a mixture of giant forms with hyperlobulated nuclei (40/40, 100%), normal and small sized maturing elements (37/40, 92.5%) and naked nuclei (17/40, 42.5%). In two cases (2/40, 5%) megakaryocytes displayed bulbous (“cloud-like”) nuclei.

Finally, in five cases (5/40, 12.5%) a mild degree of reticulin fibrosis (MF-1) was evident together with an increase of the micro-vessel density (6/40, 16%).

## Discussion

In this real-life, single-center cohort study, we investigated the mutational profiles and reviewed the clinical data and BM biopsies of 40 consecutive triple-negative ET patients with a median follow-up of 7 years.

From a strictly clinical point-of-view, we first confirmed that triple-negative ET is a very indolent disease, with a low incidence of vascular events as only two patients suffered from thrombosis. Furthermore, no case of fibrotic progression or leukemic evolution was registered, thus confirming the different behavior of this condition when compared with triple-negative PMF. Interestingly, MDS evolution was diagnosed in one (2.5%) patient after more than 32 years of follow-up, underlying therefore the need of a prompt recognition of different types of disease progression in MPNs (41).

It is worth noting that at least one non-driver nucleotide variant predicted as damaging by *in silico* tools was found in most subjects, the most frequently detected variants involving the *TET2* gene (22/40 patients, 55.0%). This finding is in line with a recent report on the occurrence and prognostic relevance of DNA sequence variants/mutations other than *JAK2/CALR/MPL* in both PV and ET (42, 43). Indeed, NGS revealed that 53% of 183 Mayo Clinic ET patients harbored one or more sequence variants/mutations other than *JAK2/CALR/MPL*, being *TET2* and *ASXL1* the most frequent ones.

Interestingly, some of these variants are reported at high VAF in the literature, in a non-negligible fraction of cases within large cohort studies focusing on the topic of triple-negative MPNs (44) and clonal hematopoiesis of indeterminate potential (CHIP) (45, 46). In accordance with these data, in our cohort we also found a high VAF, with a mean value of 43%. Interestingly, with the notable differences of thrombocytosis and a lower median age, our patients share with CHIP individuals an unremarkable clinical history, with a very indolent disease, and variants affecting genes regarded as initiator of clonal expansion (e.g., *TET2* or *ASXL1*), as well as genes with higher impact on disease progression (e.g., *RUNX1*). On the contrary, CHIP-associated *DNMT3A* variants were not recorded in our series. From a speculative point of view, in analogy with CHIP, triple-negative ET patients may constitute a very specific subset of individuals featuring low-impact laboratory abnormalities. Furthermore, it may be argued that at least some of the variants identified by our analysis may represent mere polymorphisms, without a phenotypic effect. However, we report an enhanced frequency of variants such as *TET2* c.5162T>G (p.L1721W), which features a MAF = 0.11 but it is observed in 27% of our patients, suggesting a non-casual association, though basing on low numbers. The same *TET2* variant is also predicted as damaging according to most predictive tools. Furthermore, we report a few variants, such as *RB1* c.929G>A (p.G310E), which does not represent a polymorphism (MAF = 0.0003) but features high VAF. As to the interpretation of molecular findings, we deal with the limitations of an *in silico* approach for assessment of pathogenicity or protein function damage, as we relied on databases for clinical prediction of current use in molecular diagnostics. In absence of functional studies to prove the real biological impact of such variants, their finding may be helpful to determine the clonal nature of disease in triple-negative MPNs and complement the morphological criteria (47). This stands also in accordance with the revised 2017 WHO criteria.

Thanks to the NGS approach, we could reveal the presence of non-canonical variants. Indeed, *KIT* mutations were detected in more than one third of patients (27.5%), apparently overrepresented than usually reported in MPNs, although none harbored the canonical D816V variant. Moreover, in four cases we found the c.1621A>C (M541L) variant of *KIT*, which has been shown to be associated with chronic eosinophilic leukemia, not otherwise specified (48).

Specifically concerning mutations in genes which are already well-known to be associated with poor prognosis in other MPNs (49), particularly in PMF, all *ASXL1*-mutated cases have a BM biopsy showing histological features typical for ET; with regards to the six *IDH1*-mutated subjects, one displayed BM fibrosis grade 1. As far as their biologic significance, *ASXL1* mutation is recognized within the so-called CHIP, as early initiator of a clonal expansion (50, 51) and as favoring a state of thrombocytosis (52). Furthermore, it should be highlighted that none of our cases harbored variants (affecting *TP53, SRSF2* and/or *TET2*) previously reported to impact on leukemic evolution of MPNs (53).

Interestingly, in all cases a cytogenetic analysis was performed at diagnosis and chromosomal abnormalities were detected in seven (17.5%) patients, most being, however, represented by sexual chromosome abnormalities (-Y or -X, 5/7), which cannot be considered as a marker of disease. Considering instead the two patients with the +8 detected, we can speculate that this chromosomal alteration may help in resolving clonality, even though not representing a chromosomal abnormality typical of ET, as it can be detected also in other myeloid neoplasms, including MPNs (i.e., both PMF and PV), as well as MDS or acute myeloid leukemia (40).

On the contrary, NGS provided a relevant contribution to the diagnosis of MPNs; its use should therefore be implemented in clinical practice, as it also allows the detection of novel variants.

As far as the histologic features of our series are concerned, most of the cases showed a classic ET picture characterized by a normocellular BM with an increased number of large to giant megakaryocytes with hyperlobulated nuclei forming loose clusters while about one fourth of the patients displayed a variable increase of the erythropoietic series with left-shifting and reduced myeloid/erythroid ratio. Furthermore, in about 12% of the cases a mild increment of BM fibrosis with increased micro-vessel density was identified. These histopathologic features, identified singularly, do not inhibit a clinical-pathological diagnosis of ET according to the WHO classification. However, they raise the concern of a more appropriate classification of those patients, questioning whether subjects with triple-negative, persistent thrombocytosis, but lacking the morphological features of PV or pre-fibrotic PMF, should be classified as ET or attributed to the myeloproliferative neoplasms, unclassifiable (MPN, U) category to be followed over time and classified according to the clinical evolution and any progressive BM changes (54, 55).

Finally, it is important to note that in the one single case progressed toward MDS after 32 years of follow-up, mutations of predicted pathogenic significance were found by NGS for *ASXL1* and *KIT* genes, whereas the BM morphology, alongside with the classical ET thrombocytopoiesis, displayed also features of hypercellularity and left-shifted erythropoiesis and presence of megakaryocytes with maturation defects.

We are aware that the main limitations of the present study are represented by its retrospective nature and by the relatively small sample size, preventing us from drawing any definitive conclusion on the prognostic impact of the molecular profile in this specific subgroup of patients suffering from an indolent disease. Further limits include sequencing of *JAK2* and *MPL* hotspot regions, which may certainly impair the capability of detection of novel variants of driver genes (24, 25) and the sensitivity of RQ-PCR and NGS, which may have missed mutations in driver genes at very low VAF. Finally, in absence of matched germline DNA in most of the patients, we cannot reliably rule out the alternative hypothesis of somatic vs. germline variants.

It should be stressed that in the present study a triple-negative definition was initially applied in the context of a molecular characterization performed on a routine basis: when using an extended NGS panel, the clonal nature of hematopoiesis has then been demonstrated in the majority of the cases by identifying sequence variants/mutations affecting other genes involved in myeloid malignancies. In this way, we could demonstrate that a sizeable proportion of triple-negative ET patients do have a clonal disease, thus preventing issues arising concerning their treatment, especially when a cytoreductive therapy may be warranted. Finally, our results stress the pivotal role of strict adherence to histopathologic criteria, with the prime importance given to a thorough assessment of megakaryocyte morphology, to identify those patients that, besides a formal diagnosis of triple-negative ET vs. MPN, U, can be expected to portend an indolent clinical course.

## Data Availability Statement

The original contributions presented in the study are included in the article as Supplementary Table 1. In addition, COSMIC - Catalogue Of Somatic Mutations in Cancer (RRID:SCR_002260) and dbSNP - Database of Single Nucleotide Variations (RRID:SCR_002338) were used to annotate each sequence variant.

## Ethics Statement

This study was carried out in accordance with the recommendations of Good Clinical Practice. At their first admission to our Hospital, all subjects gave informed consent to retrospectively collect clinical and laboratory data, in accordance with the Declaration of Helsinki. Accordingly, a new submission to the ethics committee for the present study was not required.

## Author Contributions

DC, GAC, UG, and AI interpreted the data and prepared the text. GAC and UG reviewed bone marrow biopsies. ST, PB, EF, and SF carried out the mutational analyses. DC, AI, CB, MB, and KB followed the patients and collected the data. All authors contributed to the article and approved the submitted version.

## Conflict of Interest

The authors declare that the research was conducted in the absence of any commercial or financial relationships that could be construed as a potential conflict of interest.
