# Supplementary material for: Triple-Negative Essential Thrombocythemia: Clinical-Pathological and Molecular Features. A Single-Center Cohort Study
Source: Front Oncol. 2021 Mar 12;11:637116. doi: 10.3389/fonc.2021.637116 (PMC8006378; doi:10.3389/fonc.2021.637116)
Supplement: Supplementary file 1 [file Data_Sheet_1.pdf]

Supplementary Material

Supplementary Table 1. List of DNA targets included in the panel

**Hotspot gene (23)**									
ABL1	*BRAF*	*CBL*	*CSF3R*	*DNMT3A*	*FLT3*	*GATA2*	*HRAS*	*IDH1*	*IDH2*
JAK2	*KIT*	*KRAS*	*MPL*	*MYD88*	*NPM1*	*NRAS*	*PTPN11*	*SETBP1*	*SF3B1*
SRSF2	*U2AF1*	*WT1*							
**Full gene (17)**									
ASXL1	*BCOR*	*CALR*	*CEBPA*	*ETV6*	*EZH2*	*IKZF1*	*NF1*	*PHF6*	*PRPF8*
RB1	*RUNX1*	*SH2B3*	*STAG2*	*TET2*	*TP53*	*ZRSR2*			

(ref. https://science-docs.illumina.com/documents/LibraryPrep/ampliseq-myeloid-panel-data-sheet-770-2018-016/Content/Source/Library-Prep/AmpliSeq/myeloid-panel/ampliseq-myeloid-panel-data-sheet.html#Introduction)

Supplementary Table 2. Description of identified variants

Gene	CDS mutation	AA change	Variant type	rs (dbSNP)	COSMIC ID	Variant classification*	MAF (dbSNP)	FATHMM-MKL	SIFT	DANN	PolyPhen-2	Sample name	Observed VAF
ASXL1	c.4189G>A	p.G1397S	missense_variant	rs146464648	COSM133033	P[1]/B[2-3]	0.0008	Damaging	Damaging	Damaging	Possibly damaging	TN_01	0.45
ASXL1	c.2595del	p.E865Dfs*2	Deletion - Frameshift	-	COSM5985563	na[1]/P[2]/LP[3]	-	na	na	na	na	TN_11	0.48
ASXL1	c.2985C>T	p.H995=	synonymous_variant	rs62206933	COSM6232766	P[1]/B[2-3]	0.009	Damaging	na	Tolerated	na	TN_33	0.52
CEBPA	c.152G>A	p.P51L	missense_variant	rs1341205888	-	na[1]/VUS[2-3]	0.000008	Damaging	Damaging	Damaging	Benign	TN_43	0.48
ETV6	c.628C>T	p.R210C	missense_variant	rs762232508	COSM3456948	P[1]/B[2]/VUS[3]	0.000076	Damaging	Tolerated	Damaging	Probably damaging	TN_42	0.49
ETV6	c.542G>A	p.R181H	missense_variant	rs150089916	-	na[1]/B[2]/VUS[3]	0.0004	Damaging	Damaging	Damaging	Probably damaging	TN_57	0.50
EZH2	c.553G>C	p.D185H	missense_variant	rs2302427	COSM3762469	P[1]/B[2-3]	0.07	Damaging	Damaging	Damaging	Benign	TN_10<br>TN_41<br>TN_43<br>TN_55<br>TN_59<br>TN_63	0.49<br>0.49<br>0.51<br>0.49<br>0.49<br>0.49
IDH1	c.315C>T	p.G105=	synonymous_variant	rs11554137	COSM1741220	P[1]/B[2-3]	0.05	Damaging	na	Tolerated	Na	TN_02<br>TN_03<br>TN_18<br>TN_23<br>TN_42<br>TN_54	0.52<br>0.49<br>0.24<br>0.49<br>0.49<br>0.27
IKZF1	c.1176C>T	p.N392=	synonymous_variant	rs61731356	COSM4162286	P[1]/B[2-3]	0.05	Damaging	na	Tolerated	Na	TN_22<br>TN_31<br>TN_42	0.49<br>0.48<br>0.50
KIT	c.1621A>C	p.M541L	missense_variant	rs3822214	COSM28026	P[1]/B[2-3]	0.07	Damaging	Tolerated	Tolerated	Benign	TN_04<br>TN_20<br>TN_22<br>TN_37	0.49<br>0.48<br>0.98<br>0.49
KIT	c.1638A>G	p.K546=	Synonym.us_variant	rs55986963	COSM21983	P[1]/B[2-3]	0.02	Damaging	na	Tolerated	Na	TN_11<br>TN_14	0.51<br>0.48
KIT	c.67+4G>A	p.?	splice_region_variant,intron_variant	rs72550820	COSM5020196	P[1]/B[2-3]	0.02	Damaging	na	Tolerated	Na	TN_14<br>TN_47	0.50<br>0.49
KIT	c.2394C>T	p.I798=	synonymous_variant	rs55789615	COSM1307	P[1]/B[2-3]	0.02	Damaging	na	Tolerated	Na	TN_18<br>TN_27<br>TN_46	0.46<br>0.50<br>0.49
RB1	c.929G>A	p.G310E	missense_variant	rs200844292	COSM327253	P[1]/B[2]/VUS[3]	0.0003	Damaging	Tolerated	Damaging	Probably damaging	TN_03<br>TN_18	0.49<br>0.26
RILP/PRPF8	c.6294G>A	p.K2098=	upstream_gene_variant, synonymous_variant	rs11559309	COSM5021164	P[1]/B[2-3]	0.04	Damaging	na	Tolerated	Na	TN_03<br>TN_09<br>TN_16<br>TN_18<br>TN_31<br>TN_35<br>TN_57	0.46<br>0.52<br>0.50<br>0.23<br>0.48<br>0.51<br>0.27
PRPF8	c.3455C>T	p.A1152V	missense_variant	-	-	na[1]/VUS[2-3]	-	Damaging	Damaging	Damaging	Probably damaging	TN_16	0.11
RUNX1	c.167T>C	p.L56S	missense_variant	rs111527738	COSM24756	P[1]/B[2-3]	0.015	Damaging	Tolerated	Tolerated	Na	TN_31<br>TN_54<br>TN_58	0.49<br>0.48<br>0.48
SH2B3	c.1421C>T	p.T474M	missense_variant	rs200567433	COSM5519182	P[1]/VUS[2-3]	0.000165	Damaging	Damaging	Damaging	Probably damaging	TN_22	0.53
STAG2	c.1108C>T	p.R370W	missense_variant	-	COSM1315173	P[1]/LP[2]/VUS[3]	-	Damaging	Damaging	Damaging	Probably damaging	TN_03	0.07
TET2	c.86C>G	p.P29R	missense_variant	rs12498609	COSM5020248	P[1]/B[2-3]	0.06	Damaging	Tolerated/Damaging	Damaging	Possibly damaging	TN_01<br>TN_09<br>TN_22<br>TN_27<br>TN_32<br>TN_63	0.48<br>0.52<br>0.52<br>0.47<br>0.48<br>0.50
TET2	c.3743T>G	p.L1248R	missense_variant	rs372179780	COSM5879020	P[1]/LP[2]/VUS[3]	-	Damaging	Damaging	Damaging	Probably damaging	TN_03<br>TN_18	0.12<br>0.06
TET2	c.5162T>G	p.L1721W	missense_variant	rs34402524	COSM5020013	P[1]/B[2-3]	0.11	Damaging	Damaging	Tolerated	Possibly damaging	TN_09<br>TN_14<br>TN_17<br>TN_19<br>TN_26<br>TN_31<br>TN_35<br>TN_41<br>TN_46<br>TN_59<br>TN_61	0.51<br>0.48<br>0.42<br>0.48<br>0.51<br>0.50<br>0.50<br>0.48<br>0.41<br>0.48<br>0.49
TET2	c.1088C>T	p.P363L	missense_variant	rs17253672	COSM5020141	P[1]/B[2-3]	0.05	Damaging	Damaging	Damaging	Benign	TN_14<br>TN_19<br>TN_26<br>TN_41<br>TN_46<br>TN_57<br>TN_61<br>TN_64	0.52<br>0.42<br>0.48<br>0.48<br>0.42<br>0.23<br>0.49<br>0.51
TET2	c.100C>T	p.L34F	missense_variant	rs111948941	COSM5941242	P[1]/B[2]/LB[3]	0.016	Damaging	Damaging	Damaging	Benign	TN_16	0.51
TET2	c.1064G>A	p.G355D		rs61744960		P[1]/LB[2]/B[3]	0.028	Damaging	Damaging	Damaging		TN_55	0.49

			missense_variant		COSM6494925						Possibly damaging	TN_64	0.49
TET2	c.3130A>T	p.K1044*	stop_gained	-	-	na[1]/VUS[2]/LP[3]	-	Damaging	na	Damaging	na	TN_26	0.03

Abbreviations: CDS, coding DNA sequence; AA, amino acid; MAF, minor allele frequency; VAF, variant allele frequency; P, Pathogenic; LP, Likely Pathogenic; VUS, Variant of Unknown Significance; LB, Likely benign; B, Benign; na, not available. * As indicated by clinical databases, such as 1: COSMIC (https://cancer.sanger.ac.uk/cosmic/), 2: Varsome (https://varsome.com/), 3: Franklin (https://franklin.genoox.com/clinical-db/home/).
